# The case for chewable tablets: reducing single-use plastic waste in pediatrics

**DOI:** 10.1017/ash.2026.10308

**Published:** 2026-02-18

**Authors:** Lydia Lu, Preeti Jaggi, Andrew Smelser, Zayd Ahmad, Suong T. Nguyen

**Affiliations:** 1 Pediatric Infectious Disease, Children’s Healthcare of Atlanta Inc, Atlanta, USA; 2 Emory University, USA; 3 Children’s Healthcare of Atlanta Inc, USA

## Abstract

Pediatric medications inflate healthcare-associated plastic waste because many are given as liquid suspensions due to a need for weight-based dosing. While chewable tablet formulations and stair-step dosing recommendations are available, they are underutilized. We demonstrate the potential of small changes to medication administration to decrease plastic waste.

## Background

An estimated 3,000 tons of plastic are discarded by US hospital systems daily, the majority as single-use plastics (SUP).^1–3^ Plastics contain chemicals potentially harmful to human health and are sourced from oil and gas. When plastics are disposed of in landfills, they emit methane, a greenhouse gas which contributes to climate change. Not all plastics can be avoided, but many practices can be modified to safely decrease SUP use.

Oral medications for young children are often given as a liquid suspension for ease of dosing and administration but require syringes and cups to administer. However, many common medications can be given in chewable tablet formulations, which can be dosed by weight bracket, do not need special containers for administration, are easier to store, and less prone to waste. We sought to quantify the amount of SUP waste created during hospitalizations with administration of the three most prescribed pediatric medications for which tablet formulations are available: acetaminophen, ibuprofen, and amoxicillin, the most frequently prescribed pediatric antibiotic.

## Methods

Children’s Healthcare of Atlanta is a pediatric hospital system with three free-standing hospitals. We explored Epic electronic medical records (electronic version 35.7.2) deidentified data using Slicer Dicer, an embedded data exploration tool, to inventory all oral acetaminophen, ibuprofen, and amoxicillin doses given to pediatric patients from 2 to 21 years of age in 2024 by our hospital pharmacies and categorized them by liquid versus tablet formulation. We weighed the SUP items utilized to dispense these medications to quantify plastic waste. Medication costs were obtained from UpToDate Lexidrug (accessed October 17, 2025), and purchase costs of wholesale plastic dispensing devices were obtained from our hospital pharmacy.

The impact of the SUP waste created by dispensing these three medications (acetaminophen, ibuprofen, and amoxicillin) for one year in our hospital system was estimated using the conversion factor of 3.10 kg of CO_2_ equivalent emissions per 1 kg of plastic waste, per the UK Centre for Sustainable Healthcare.^4^

## Results

According to standard operating procedures, liquid formulations of oral acetaminophen and ibuprofen are dosed by weight and drawn into SUP syringes. Manufacturer-predosed plastic cups are batched in 10 cups per plastic storage tray and stocked in automated medication dispensing machines in hospital units. When ordered, nurses access the cups, draw out the appropriate dose by plastic syringe, and dispense the medication to the patient. Unused drug is wasted. Amoxicillin liquid doses are drawn up by the pharmacy in plastic syringes, labeled, secured in individual plastic bags, and sent to patient care areas where the syringe is used to administer the medication. In contrast, chewable acetaminophen and ibuprofen tablets are re-packaged into a paper and plastic film blister, then stocked in hospital units. Chewable amoxicillin tablets are larger and re-packaged by the pharmacy into plastic and paper blister packs. Nurses obtain acetaminophen and ibuprofen tablets from the automated medication machines and transfer the appropriate dose from the blister pack to a plastic medication cup for presentation to patients. Amoxicillin tablets blister packs are sent from the pharmacy in small plastic bags to hospital units where the medication is transferred to a plastic medication cup for administration. Photos of the wasted plastic and the estimated plastic waste per dose of drug by formulation are summarized in Supplemental Table 1. Accounting for the cost of plastic dispensing devices and the cost per dose of medication, liquid acetaminophen and ibuprofen cost about $.10 more than tablets per dose. However, chewable amoxicillin, at $.74 per 250 mg tablet, costs $.32 more per administration than an equivalent liquid dose. (Supplemental Table 2)

In 2024, 86,216 doses of acetaminophen, 74,100 doses of ibuprofen, and 11,368 doses of amoxicillin were dispensed to patients 2–21 years old. Of these, 61.6% (*n* = 53,135) of acetaminophen, 68.4% (*n* = 50,656) of ibuprofen, and 76.1% (*n* = 8,652) of amoxicillin doses were given in a liquid formulation. For preparation of liquid medication doses, the estimated total weight of the plastic used was 1,017 kg, equivalent to 3,367 kg of CO_2e_ emissions. In contrast, the estimated total weight of the plastic associated with tablet dispensing was 64.4 kg, equivalent to 213 kg of CO_2e_ emissions for the same period (Table 1). Overall, the amount of plastic used per dose of liquid medication was 9 times that used for each tablet dose.

**Table 1.** Plastic waste in 2024 per drug by formulation

	Acetaminophen 86,216 doses	Ibuprofen 74,100 doses	Amoxicillin 11,368 doses	Totals
Liquid doses	53,135 (61.6%)	50,656 (68.4%)	8,652 (76.1%)	112,443 doses
Plastic waste per liquid dose	9.3g	9.3g	6g	
Total plastic waste for liquid doses	494,156g	471,101g	51,912g	1,017,168g
Tablet doses	33,081 (38.4%)	23,444 (31.6%)	2,716 (23.9%)	59,241 doses
Plastic waste per tablet dose	1g	1g	2.9g	
Total plastic waste for tablet doses	33,081g	23,444g	7,876g	64,401g

**Figure 1.**
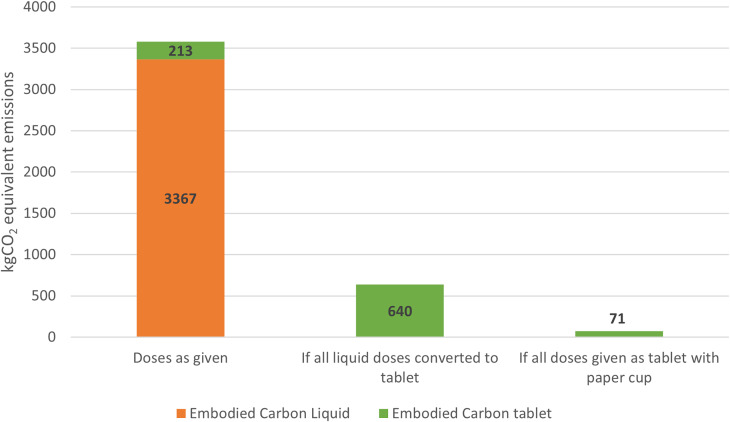
Embodied carbon emissions associated with plastic versus tablet medication dosing.

If all liquid doses of amoxicillin, ibuprofen, and acetaminophen had been given as tablets, a total 823 kg of plastic waste, equivalent to 2,727 kg of CO_2e_ emissions, could have been spared. If paper cups were used instead of plastic cups for dispensing tablets, an additional 171 kg of plastic waste, or 568 kg of CO_2e_ emissions could have been spared, taking the total CO_2e_ emissions generated from dosing these three medications in one year from 3,367 kg CO_2e_ to 71 kg CO_2e_ (Figure 1). The combined 3,296 kg CO_2e_ emissions saving potential is approximately equivalent to driving 9,921 miles in a gas-powered passenger vehicle, the distance from New York to Tokyo, crossing the Pacific Ocean.^5^

## Conclusions

Despite the availability of chewable tablet formulations and stair-step dosing recommendations for acetaminophen, ibuprofen, and amoxicillin, these medications were primarily administered to our patients in liquid formulations, generating substantially more SUP waste than tablet administration. Strategies to decrease the need for SUP for medication administration should be explored. Foremost, reducing low-value tests and therapies consistent with the Choosing Wisely campaign would decrease unnecessary medication orders.^6^ When needed, paper cups to administer tablets could be used instead of plastic. Prestocking frequently utilized medication at the point of care would further decrease the need for plastic to transport medication from pharmacies. Medical systems can reduce spending on SUP by strategically considering their utilization of SUP for medication administration and updating their standard operating procedures. Educating providers and caregivers on tablet availability increases oral treatment options with little downside but with clear potential to reduce waste, including medication waste. Leveraging the suggestive power of appropriate electronic order sets to prompt consideration for IV-to-oral conversion of medications, non-liquid medication formulations, automatic stop times, and discontinuation of medication orders as part of discharge planning can further support waste reduction goals.^9^

We used a simple gate-to-grave exercise, rather than a more comprehensive cradle-to-grave life cycle analysis which would have accounted for additional differences in shipping, manufacturing, storage, and landfill considerations for tablets vs liquid medication.^7^ However, even with our likely underestimation of impact, the opportunity to incorporate small changes and send less SUP to landfills is compelling. Additional targets for waste reduction include plastic associated with purchasing and stocking of medications. In practice, elevating tablets as a standard choice may be challenged by patients and parents who are accustomed to the palatability of certain syrups, and optimizing the acceptability of chewable tablets to children should be explored. Specific situations also merit further consideration such as with antiretroviral medications where pharmacokinetics differ by pediatric formulation.^8^ This exercise evaluated the potential benefit with adjusting our practices for three common medications; the true potential is in widely propagating this approach to all medications.

## Supporting information

10.1017/ash.2026.10308.sm001 Lu et al. supplementary material Lu et al. supplementary material

## Supplementary material

The supplementary material for this article can be found at https://doi.org/10.1017/ash.2026.10308.
