# Supplementary material for: The case for chewable tablets: reducing single-use plastic waste in pediatrics
Source: Antimicrob Steward Healthc Epidemiol. 2026 Feb 18;6(1):e50. doi: 10.1017/ash.2026.10308 (PMC12936800; doi:10.1017/ash.2026.10308)
Supplement: Lu et al. supplementary material [file S2732494X26103088sup001.docx]

**Case for Chewable Tablets: Supplemental Submission**

**Supplemental Table 1: Plastic waste by medication administration**

|  |  |  | Acetaminophen | | Ibuprofen | | Amoxicillin | |
| --- | --- | --- | --- | --- | --- | --- | --- | --- |
|  |  |  | Liquid  (g) | Tablet  (g) | Liquid  (g) | Tablet  (g) | Liquid  (g) | Tablet  (g) |
| Plastic syringe | 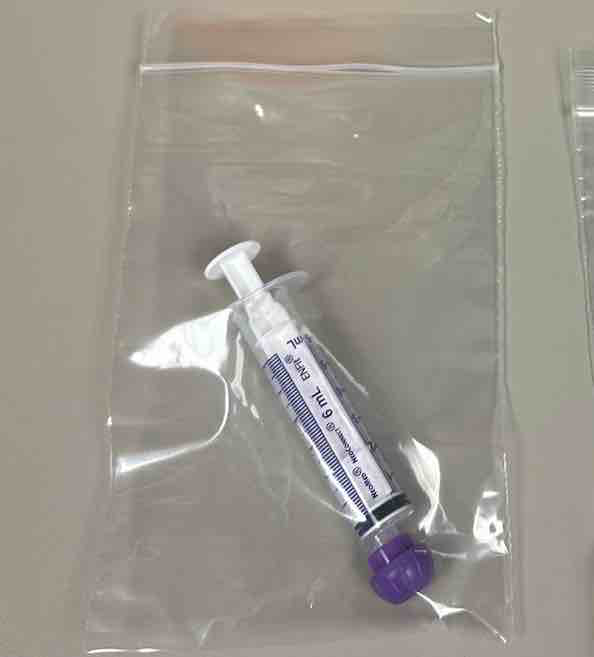 |  | 4.4 | NA | 4.4 | NA | 4.4 | NA |
| Tray + pre-packaged cup | 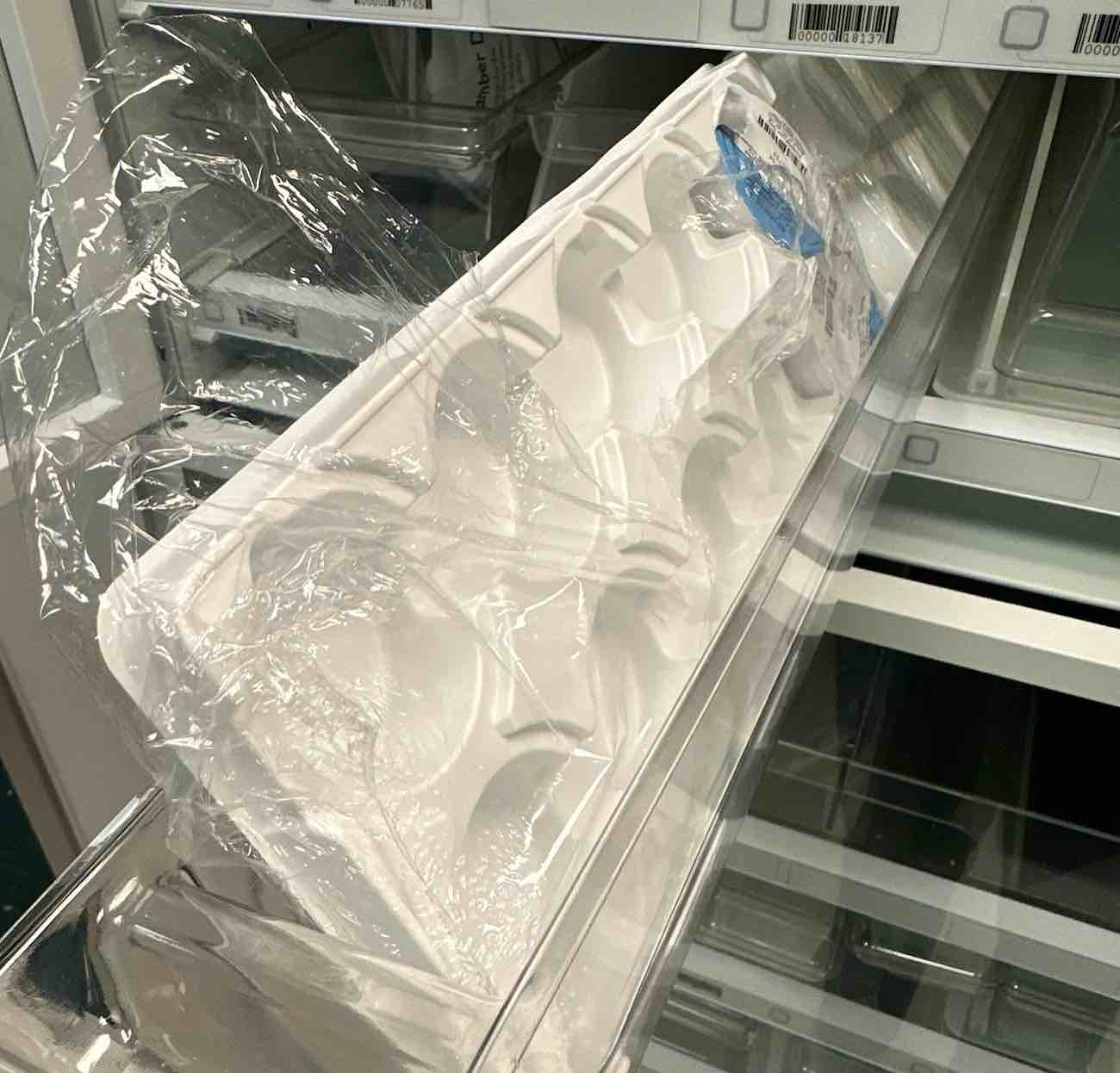 | 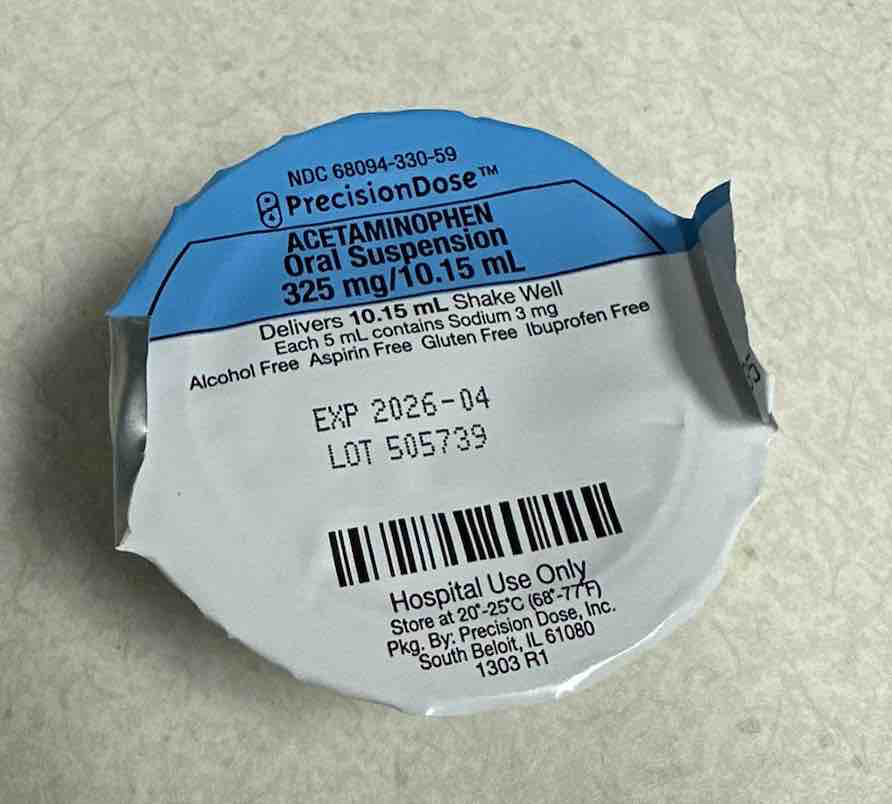 | 4.9 | NA | 4.9 | NA | NA | NA |
| Blister pack | 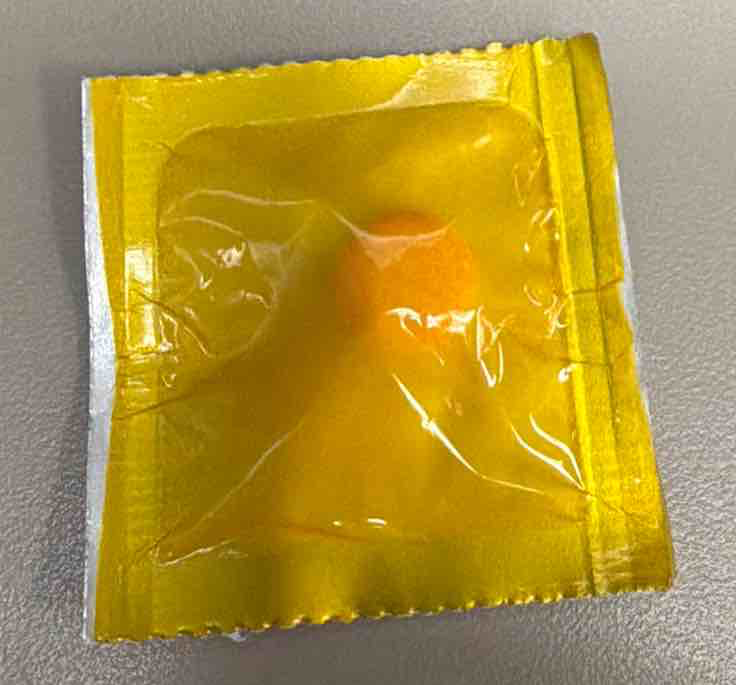 | 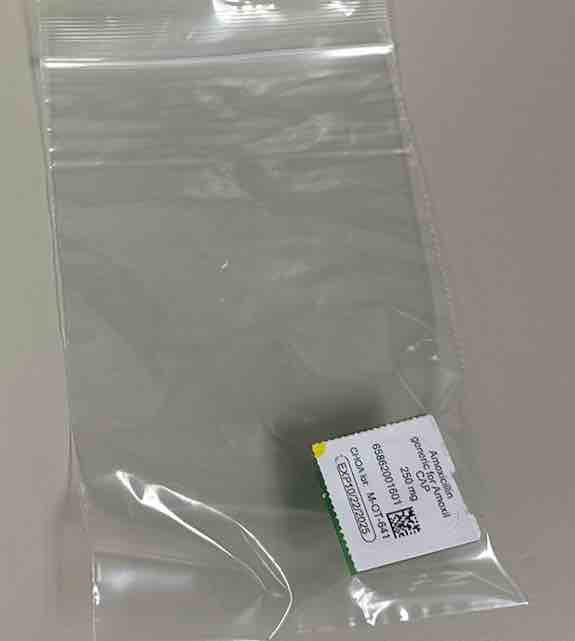 | NA | negligible | NA | negligible | NA | 0.3 |
| Plastic bag | 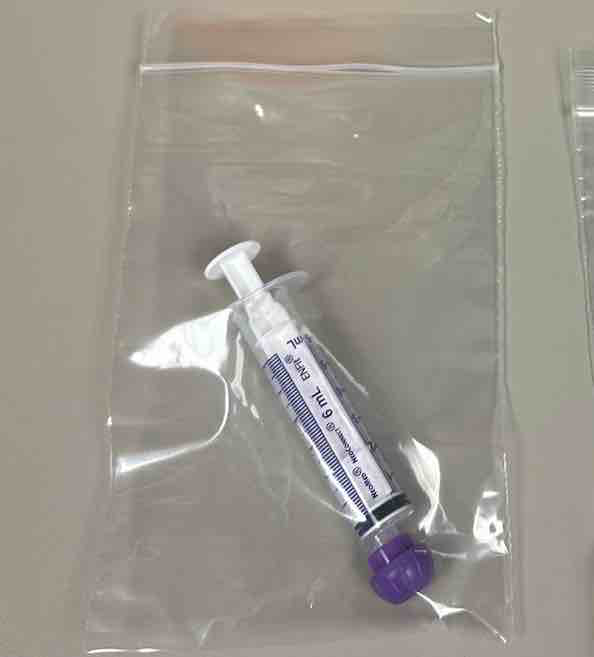 | 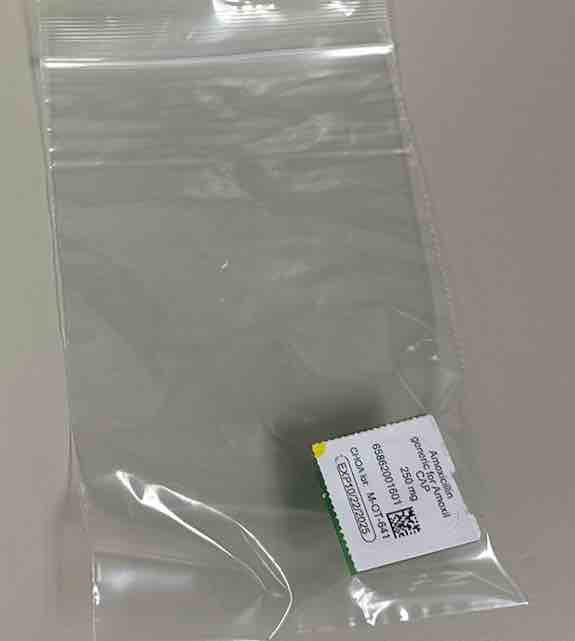 | NA | NA | NA | NA | 1.6 | 1.6 |
| Plastic cup | 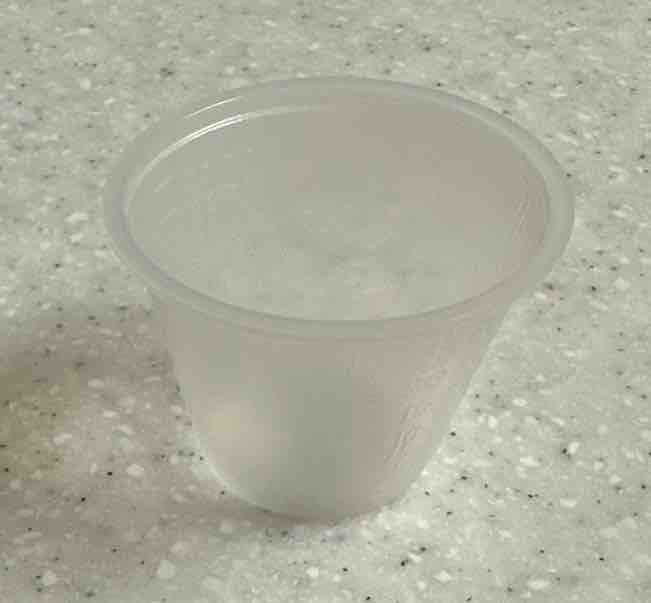 |  | NA | 1 | NA | 1 | NA | 1 |
| Total plastic per administration |  |  | 9.3 | 1 | 9.3 | 1 | 6 | 2.9 |

**Supplemental Table 2: Cost by medication administration**

|  | Acetaminophen | | Ibuprofen | | Amoxicillin | |
| --- | --- | --- | --- | --- | --- | --- |
|  | Liquid | Tablet | Liquid | Tablet | Liquid | Tablet |
| Dosing | 160mg/5mL | 160mg | 100mg/mL | 100mg | 400mg/5mL | 250mg |
| Cost of drug ($) | 0.30 | 0.29 | 0.30 | 0.29 | 0.31 | 0.74 |
| Cost of syringe ($) | 0.11 | NA | 0.11 | NA | 0.11 | NA |
| Cost of plastic cup ($) | NA | 0.01 | NA | 0.01 | NA | 0.01 |
| Cost of plastic bag ($) | NA | NA | NA | NA | unknown | unknown |
| Total cost per administration ($) | 0.41 | 0.30 | 0.41 | 0.30 | 0.42 | 0.75 |
|  |  |  |  |  |  |  |
